# Transitions between Liquid Crystalline Phases Investigated by Dielectric and Infrared Spectroscopies

**DOI:** 10.1021/acs.jpcb.5c07310

**Published:** 2026-01-28

**Authors:** Aleksandra Deptuch, Natalia Osiecka-Drewniak, Anna Paliga, Natalia Górska, Anna Drzewicz, Katarzyna Chat, Mirosława D. Ossowska-Chruściel, Janusz Chruściel

**Affiliations:** † Institute of Nuclear Physics Polish Academy of Sciences, Radzikowskiego 152, PL-31342 Kraków, Poland; ‡ Faculty of Physics, Astronomy and Applied Computer Science, 37799 Jagiellonian University, Łojasiewicza 11, PL-30348 Kraków, Poland; § Faculty of Chemistry, Jagiellonian University, Gronostajowa 2, PL-30387 Kraków, Poland; ∥ Faculty of Science, University of Siedlce, 3 Maja 54, PL-08110 Siedlce, Poland

## Abstract

The liquid crystalline 11OS5 compound, forming the nematic phase and a few smectic phases, is investigated by broadband dielectric spectroscopy and infrared spectroscopy. The dielectric relaxation times, ionic conductivity, and positions of infrared absorption bands corresponding to selected intramolecular vibrations are determined as a function of temperature in the range from an isotropic liquid to a crystal phase. The correlation coefficient matrix and k-means cluster analysis of infrared spectra are tested for detection of phase transitions. The density-functional theory calculations are carried out for interpretation of experimental infrared spectra. The performance of various basis sets and exchange–correlation functionals is compared, including both agreement of scaled calculated band positions with experimental values and computational time. The intermolecular interactions in the crystal phase are inferred from the experimental IR spectra and density-functional theory calculations for dimers in head-to-head and head-to-tail configurations. The experimental temperature dependence of the C=O stretching band suggests that the head-to-tail configuration in the crystal phase is more likely. A significant slowing down of the flip-flop relaxation process is observed at the transition between the smectic C and hexagonal smectic X phases.

## Graphical abstract

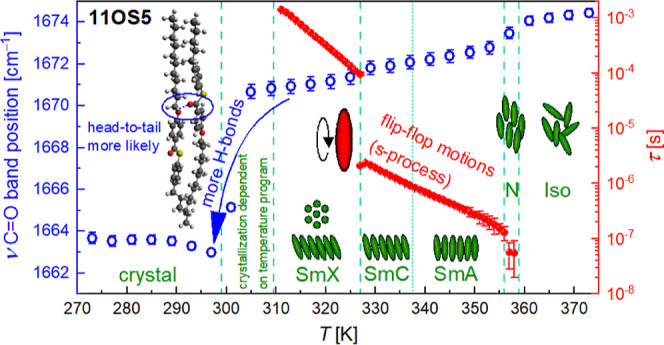

## 1 Introduction

Broadband dielectric spectroscopy (BDS) gives insight into the relaxation processes of dipoles in a weak, alternating electric field.^1^ In liquid crystals, it involves molecular processes – rotations of molecules around their short and long axes and, in some cases, also collective processes – fluctuations of order parameters.^2^ The BDS method is applied for identification of liquid crystalline phases,^3–5^ determination of phase transition temperatures,^5–7^ as well as investigation of the glass transition^5,8^ and crystallization kinetics.^8,9^ Complementary infrared (IR) spectroscopy reveals the intramolecular vibrations, which change a molecule’s dipole moment.^10^ This method is applied for investigation of the sample’s purity,^11,12^ molecular conformation,^13,14^ and intermolecular interactions.^14,15^ Some IR absorption bands are sensitive to phase transitions, especially those related to significant changes in structure, like melting of a crystal.^14,15^

The purpose of this study is the analysis of the dielectric and IR spectra of 4-pentylphenyl-4′-undecyloxythiobenzoate, denoted as 11OS5 (Figure 1), focused on transitions between the mesomorphic and crystal phases of this compound. 11OS5 forms several thermotropic liquid crystalline phases between the isotropic liquid (Iso) and crystal (Cr):^16^

- Nematic (N) with a long-range orientational order of long molecular axes.^17^
- Smectic A (SmA) with a quasi-long-range lamellar order and zero average tilt of molecules within smectic layers.^17^
- Smectic C (SmC) with quasi-long-range lamellar order and nonzero average tilt of molecules within smectic layers.^17^ The tilt angle in the SmC phase of 11OS5, estimated from the X-ray diffraction patterns, reaches only 11°.^16^
- Smectic X (SmX), a crystal-like tilted smectic phase with a long-range positional order described by a monoclinic unit cell and a hexagonal intralayer order. It is either the SmG phase with the unit-cell parameters *b* < *a* < *c*, α = γ = 90°, β≠90° or the SmJ phase with *a* < *b* < *c*, α = γ = 90°, β≠90°.^17^ The X-ray diffraction data from ref 16 do not enable distinction between these phases, which differ only by the direction of a molecular tilt with respect to a hexagonal ordering within layers.^17^ The tilt angle of molecules in the SmX phase of 11OS5 is equal to ca. 15°.^16^
- Smectic Y′ and Y, crystal-like smectic phases, with a long-range positional order described by the monoclinic unit cells and probably a herringbone intralayer order.^17^ The X-ray diffraction patterns of these phases were not obtained due to quick crystallization.^16^

**Figure 1:**
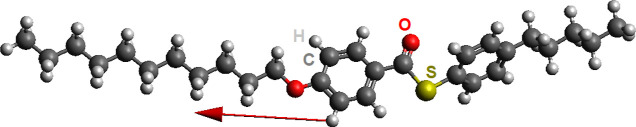
11OS5 molecular model and its dipole moment (2.4 D) optimized by the DFT method (def2TZVPP basis set, B3LYP-D3­(BJ) functional).

The phase sequence of 11OS5 during cooling at 5 K/min is Iso (357.4 K), N (355.9 K), SmA {338 K}, SmC (326.0 K), SmX (298.5 K), SmY’ (296.0 K), and SmY (294.7 K) Cr, where temperatures in () were determined by differential scanning calorimetry and the temperature in {} was determined by polarizing optical microscopy because the SmA → SmC transition was not visible in the calorimetric results.^16^ The SmX, SmY′, and SmY phases are metastable, and they are observed only after supercooling below the melting temperature of a crystal, which is 334 K.^16^ However, 11OS5 remains in the metastable SmX phase long enough to enable conducting measurements in isothermal conditions before crystallization occurs.^16^

In the first part, the relaxation times, dielectric strength, and ionic conductivity are obtained as a function of temperature from the BDS spectra of 11OS5 collected on cooling. In the second part, the experimental IR spectrum of 11OS5 in a crystal phase at room temperature is analyzed based on theoretical spectra calculated at various levels of theory. The experimental IR absorption bands are assigned to particular intramolecular vibrations, and performances of different levels of theory (scaling coefficients, root-mean square error, computational times) are compared. In the third part, the IR spectra of 11OS5 collected on cooling are analyzed using the matrix of correlation coefficients^18^ and the k-means cluster method.^19–21^ Positions of selected IR absorption bands are investigated as a function of temperature. The main aim is an attempt to detect subtle changes in the BDS and IR spectra related to the SmA → SmC transition, as well as to investigate more significant changes related to the SmC → SmX transition. The comparison of BDS and IR data may enable correlation of dielectric relaxation with intramolecular vibrations for a better understanding of molecular rearrangements during phase transitions between mesophases and a crystalline state.

## 2 Experimental and Computational Details

The synthesis of 4-pentylphenyl-4′-undecyloxythiobenzoate, abbreviated as 11OS5, is described elsewhere.^22^ The IR results presented in this work confirm the molecular structure.

The BDS measurements were performed using the Novocontrol Technologies spectrometer for a sample with a thickness of 75 μm, placed between two golden electrodes with a polytetrafluoroethylene spacer. The dielectric spectra were collected on cooling from 373 to 273 K, in the frequency range of 0.1–10^7^ Hz. The data were analyzed by fitting the complex model of the dielectric permittivity vs frequency in OriginPro.

The Fourier-transform IR measurements were performed using a Bruker VERTEX 70v FT-IR spectrometer equipped with an Advanced Research System DE-202A cryostat and ARS-2HW compressor. The IR spectra were collected by the attenuated total reflection method for a pure sample at room temperature in the wavenumber range of 370–4000 cm^–1^ and by the transmission method for a sample mixed with KBr on cooling from 373 to 273 K in the wavenumber range of 450–4000 cm^–1^, both with a resolution of 2 cm^–1^.

The correlation coefficient matrix of IR spectra collected at different temperatures was calculated using a Python script based on the SciPy and NumPy libraries.^23^ The fitting of pseudo-Voigt peak functions with a linear background to selected absorption bands and k-means cluster analysis with the Euclidean metrics^19^ were carried out in OriginPro. Background subtraction was performed before calculation of the correlation coefficient matrix and k-means cluster analysis.

The assignment of the IR absorption bands was based on the theoretical IR spectra calculated by the DFT method at various levels of theory for an isolated molecule in Gaussian 16, Revision C.01.^24^ The tested basis sets were def2SVP, def2SVPP, def2TZVP, def2TZVPP,^25,26^ 631+Gd, and 6311+Gdp.^27–30^ The applied exchange–correlation functionals were either BLYP-D3­(BJ) or B3LYP-D3­(BJ).^31–33^ The contributions of vibrational modes were calculated via the potential energy distribution analysis in VEDA.^34,35^

## 3 Results and Discussion

### 3.1 Dielectric Spectra versus Temperature

The BDS spectra of 11OS5 (Figure 2) were fitted with the complex function including the Cole–Cole model of relaxation processes, contribution from conductivity in the imaginary part ε″, and electrode polarization contribution in the real part ε′ of dielectric permittivity ε* ^2,36^

1 $$\varepsilon^{*}\left(f\right)=\varepsilon^{\prime }\left(f\right)-i\varepsilon^{\prime \prime }\left(f\right)=\varepsilon_{\infty }+\sum_{j}\frac{{\Delta \varepsilon }_{j}}{1+{\left(2\pi if\tau_{j}\right)}^{1-a_{j}}}-\frac{is_{1}}{{\left(2\pi f\right)}^{n_{1}}}+\frac{is_{2}}{{\left(2\pi f\right)}^{n_{2}}}$$

**Figure 2:**
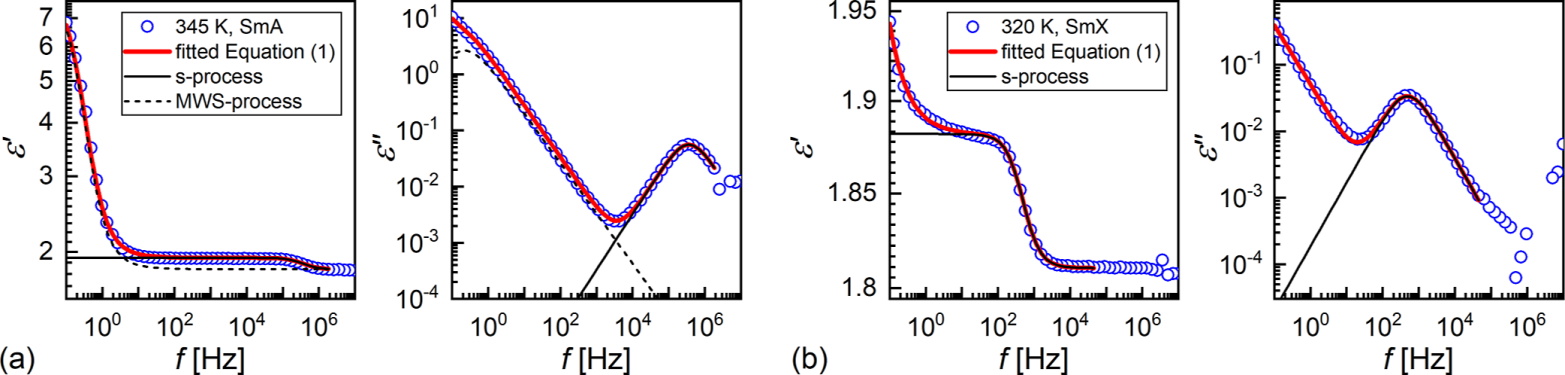
Exemplary BDS spectra of 11OS5 in the SmA (a) and SmX (b) phases with the fitting results of eq 1.

The Cole–Cole model describes each relaxation process by the dielectric strength Δε, relaxation time τ, and parameter *a*, corresponding to the distribution of the relaxation time (for the Debye model, *a* = 0); ε_∞_ is the dielectric dispersion in the limit of high frequencies; *s*_1_, *n*_1_ and *s*_2_, *n*_2_ are fitting parameters of the background at low frequencies, originating from the sample’s conductivity and electrode polarization, respectively, or from the tails of low-frequency relaxation processes. The BDS results are summarized in Figure 3. The phase transitions are visible as significant discontinuities in the temperature dependence of the dielectric dispersion, registered at ca. 1 kHz. The exception is the SmA → SmC transition, which corresponds to a very small step in the ε′(1 kHz,*T*) plot. The hexagonal SmX, despite being metastable, is observed in a temperature range wider than 15 K, while the SmY′ and SmY phases are not detected, and the direct SmX → Cr transition occurs.

**Figure 3:**
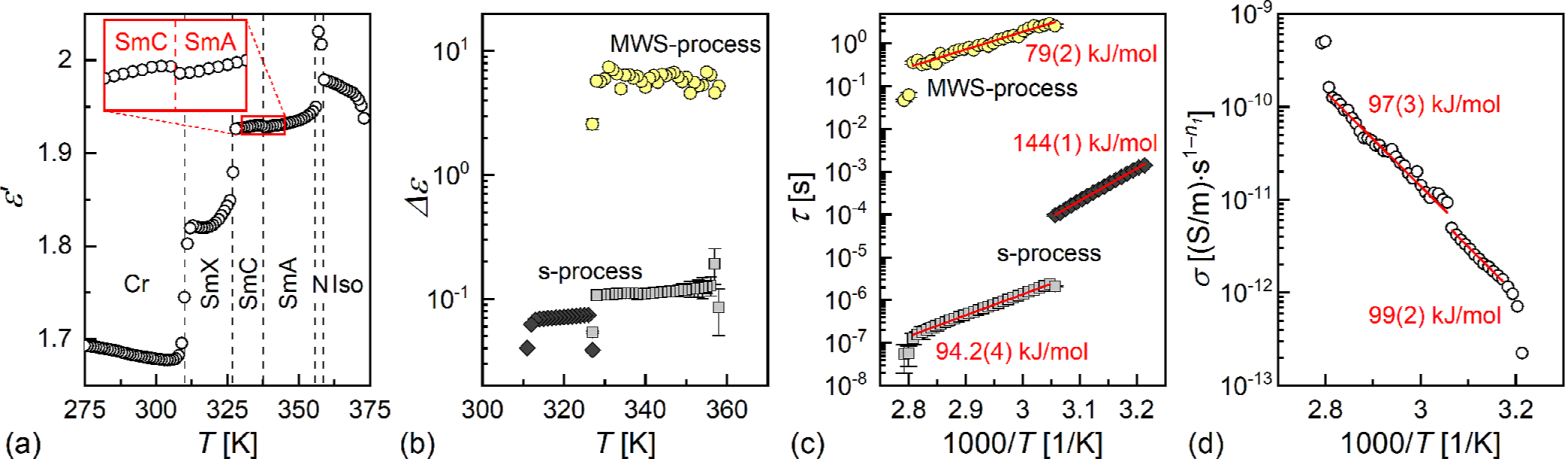
Dielectric dispersion at 1 kHz (a) and dielectric strength of relaxation processes in 11OS5 (b) as a function of temperature; activation plots of the relaxation times (c) and conductivity (d).

Two relaxation processes are observed in the isotropic liquid, nematic, and SmA and SmC phases. The fitting results of eq 1 are presented only for temperatures below the Iso → N transition because in the isotropic liquid, some of the fitting parameters had too large uncertainties. The low-frequency process, with the average Δε = 5.9, *a* = 0.11, and activation energy *E*_*a*_ = 79(2) kJ/mol, is interpreted as the Maxwell–Wagner–Sillars process.^37^ The high-frequency process, with the average Δε = 0.11, *a* = 0.01, and activation energy *E*_*a*_ = 94.2(4) kJ/mol, is the molecular s-process, originating from rotations of molecules around their short axes (called also flip-flop motions).^6,38,39^ The presented activation energies were obtained in the SmA and SmC phases. For the N phase, which has a narrow temperature range, only two BDS spectra were collected, and the activation energy was not determined. The conductivity contribution in the low-frequency region of ε″ can be fitted with an assumption of ionic conductivity, where *n*_1_ = 1.^40^ The conductivity determined as σ = *s*_1_ε_0_, where ε_0_ is the vacuum permittivity, shows a linear dependence in the activation plot, with *E*_*a*_ = 97(3) kJ/mol. The polarization contribution in the nematic phase is described by *n*_2_ = 1.5, in agreement with ref 40, while in SmA and SmC, this contribution was fixed to zero. The activation energies of the s-process obtained earlier for the SmA and SmC phases in nOS5 homologues with *n* = 8, 9, 10 are in the 92–110 kJ/mol range,^41^ comparable to the result for 11OS5.

In the hexagonal SmX phase, the s-process shifts abruptly to lower frequencies. The ratio of τ in the SmX and SmC phases at the SmC → SmX transition is equal to 46. The dielectric strength of the s-process decreases to Δε = 0.07, and the activation energy increases to 144(1) kJ/mol. The observed changes are caused by the rise of the positional order within the smectic layers. The Cole–Cole parameter equals, on average, *a* = 0.04; thus, the s-process still has an almost ideal Debye character. The conductivity part cannot be correctly described under the assumption of *n*_1_ = 1. The fitted values of *n*_1_ are equal to 0.8–0.9 and only shortly before crystallization, *n*_1_ = 0.4. The activation energy of σ, which is an effective conductivity only if *n*_1_ ≠ 1, is equal to 99(2) kJ/mol. Thus, the SmC → SmX transition leads to a decrease in conductivity, but *E*_*a*_ is the same within uncertainties. The polarization contribution is also no longer described by *n*_2_ = 1.5, as it was in the nematic phase. Instead, *n*_2_ gradually decreases on cooling from 1.1 to 0.6. Deviation from *n*_2_ = 1.5 may be caused by Maxwell–Wagner–Sillars relaxation, which is out of the measured frequency range, but the high-frequency tail still affects the collected spectra.

The earlier dielectric data for a shorter 9OS5 homologue show an increase of τ of the s-process 13 times at the SmC → SmX transition and further 1.3 times at the SmX → SmY transition.^42^ The first increase in τ is presented as occurring within the SmC phase, and SmX is not mentioned, but the recent results^16^ indicate that it was actually the SmX → SmY transition.

### 3.2 Scaling of Calculated Infrared Spectra

The assignment of IR absorption bands to intramolecular vibrations was performed for the IR spectrum collected in the reflection mode at room temperature for 11OS5 in a crystal phase (Figure 4). The theoretical band positions and intensities were calculated using the triple-ζ basis sets def2TZVPP and def2TZVP; split-valence basis sets def2SVPP and def2SVP; and 631+Gd and 6311+Gdp basis sets with diffuse corrections. The Becke exchange functional and Lee–Yang–Parr correlation functional were applied as BLYP and B3LYP functionals together with Grimme’s 3D dispersion and Becke-Johnson damping (see Section 2 for references). The calculated spectra are shown in Figure 4 below the experimental result. The band assignments as well as the comparison of experimental and calculated band positions are gathered in Tables S1–S8 in the Supporting Information (SI).

**Figure 4:**
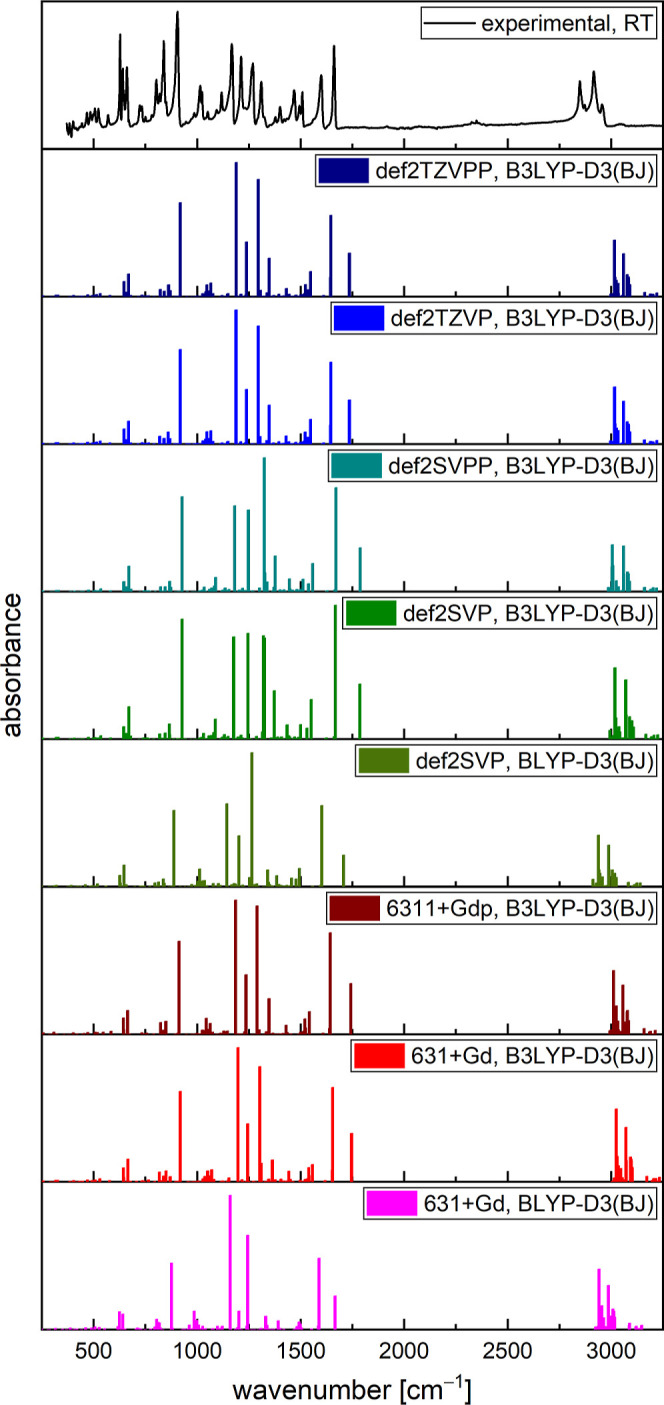
Experimental IR spectrum of 11OS5 in a crystal phase at room temperature compared to unscaled spectra calculated by the DFT method at different levels of theory.

The scaling coefficient *a* relates to experimental ${\mathrm{\nu ̃}}_{\exp}$ and calculated ${\mathrm{\nu ̃}}_{calc}$ band positions as follows: ${\mathrm{\nu ̃}}_{\exp}=a{\mathrm{\nu ̃}}_{calc}$ . It is determined as the slope of the linear fit to the ${\mathrm{\nu ̃}}_{\exp}\left({\mathrm{\nu ̃}}_{calc}\right)$ plot, with the intercept fixed to zero (Figure S1 in Supporting Information). The fitting can be performed in the full wavenumber range. However, it is advised to determine it separately for the <1000 cm^–1^, 1000–2000 cm^–1^, and >2000 cm^–1^ ranges.^43^ The results for 11OS5 (Table 1) show that the scaling coefficients at the same level of theory are larger and closer to 1 for the bands located below 2000 cm^–1^ than for those at higher wavenumbers. The scaling coefficients obtained for <1000 cm^–1^ and 1000–2000 cm^–1^ have close values, in some cases even equal within the uncertainty. In all cases where the B3LYP-D3­(BJ) functional was applied, *a* < 1, which means that calculated band positions are overestimated. The BLYP-D3­(BJ) functional leads to underestimation of band positions only below 2000 cm^–1^, *a* > 1, while the linear fit performed at higher wavenumbers and in the whole range gives *a* < 1.

**Table 1:** Scaling Factors between Experimental and Calculated IR Band Positions Determined for 11OS5.^a^

table	basis set	functional	scaling factor				RMSE [cm^–1^]	CPU time [h]
			<1000 cm^–1^	1000–2000 cm^–1^	>2000 cm^–1^	all		
S1	def2TZVPP	B3LYP-D3(BJ)	0.974(4)	0.974(2)	0.952(2)	0.961(2)	11.6	156.9
S2	def2TZVP	B3LYP-D3(BJ)	0.975(4)	0.974(2)	0.952(2)	0.962(2)	11.9	85.8
S3	def2SVPP	B3LYP-D3(BJ)	0.966(5)	0.960(4)	0.952(2)	0.952(2)	17.1	4.7
S4	def2SVP	B3LYP-D3(BJ)	0.969(5)	0.965(4)	0.950(2)	0.957(2)	17.8	6.6
S5	def2SVP	BLYP-D3(BJ)	1.005(3)	1.003(3)	0.975(2)	0.988(3)	13.6	4.7
S6	6311+Gdp	B3LYP-D3(BJ)	0.978(4)	0.975(3)	0.953(2)	0.963(2)	13.1	40.7
S7	631+Gd	B3LYP-D3(BJ)	0.977(4)	0.966(3)	0.947(2)	0.957(2)	12.5	17.6
S8	631+Gd	BLYP-D3(BJ)	1.014(4)	1.004(4)	0.974(2)	0.988(3)	15.1	8.8

a The first column refers to the detailed IR band assignments shown in SM. The root mean square error (RMSE) was calculated for experimental and scaled calculated band positions using the scaling factors obtained separately for the <1000 cm^–1^, 1000–2000 cm^–1^, and >2000 cm^–1^ ranges. The CPU times were taken from the Gaussian^24^ output files.

The root-mean square error between the experimental and scaled calculated band positions was obtained as $RMSE=\sqrt{\frac{1}{n}\sum_{i=1}^{n}{\left({\mathrm{\nu ̃}}_{\exp i}-a{\mathrm{\nu ̃}}_{calci}\right)}^{2}}$ , where *n* is the number of bands. Different values of *a* were used in the <1000 cm^–1^, 1000–2000 cm^–1^, and >2000 cm^–1^ ranges, according to Table 1. The overall scaling factor (column ‘all’ in Table 1) was not used in the calculation of RMSE. The smallest RMSE values are 11.6 cm^–1^ for def2TZVPP/B3LYP-D3­(BJ) and 11.9 cm^–1^ for def2TZVP/B3LYP-D3­(BJ). The corresponding computational times are 156.9 and 85.8 h, respectively. Thus, the longest def2TZVPP/B3LYP-D3­(BJ) calculations do not give a significant improvement in RMSE compared to twice as short def2TZVP/B3LYP-D3­(BJ) calculations. The third smallest RMSE, 12.5 cm^–1^, is obtained for 631+Gd/B3LYP-D3­(BJ), with a relatively short computational time, 17.6 h. Interestingly, much longer 6311+Gd/B3LYP-D3­(BJ) calculations lasting 40.7 h give a slightly worse RMSE equal to 13.1 cm^–1^. One of the shortest is def2SVP/BLYP-D3­(BJ) calculations, lasting 4.7 h, and the corresponding RMSE is 13.6 cm^–1^. The BLYP-D3­(BJ) functional shows a worse performance when used with the 631+Gd basis set because the computational time is longer (8.8 h) and RMSE is larger (15.1 cm^–1^) than for the def2SVP basis set. The largest RMSE values are 17.1 cm^–1^ for def2SVPP/B3LYP-D3­(BJ) and 17.8 cm^–1^ for def2SVP/B3LYP-D3­(BJ), with the computational times 4.7 and 6.6 h, respectively.

### 3.3 Infrared Spectra versus Temperature

The IR spectra of 11OS5 were collected on cooling from 373 to 273 K in the transmission mode (Figure 5). The spectra in the isotropic liquid, nematic, and smectic phases are very similar, and only crystallization shows as a sharpening of absorption bands. Three methods are applied to search for the subtle differences in IR spectra at higher temperatures: (1) calculation of the correlation coefficient matrix, (2) k-means cluster analysis, and (3) determination of the selected absorption bands’ positions as a function of temperature.

**Figure 5:**
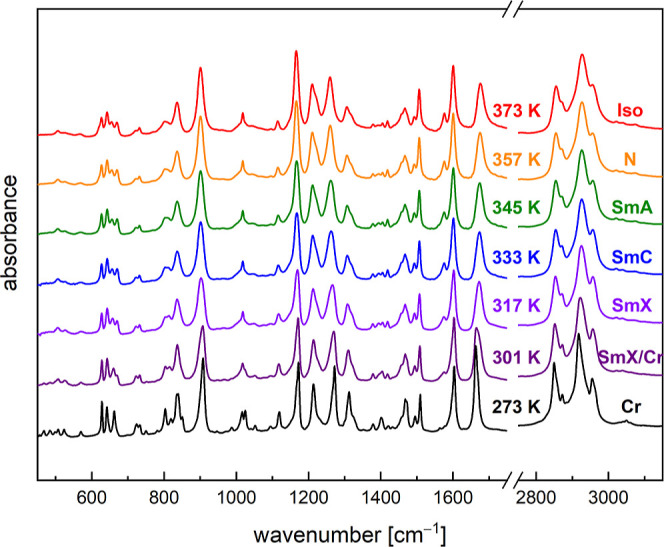
Representative experimental IR spectra of 11OS5 collected on cooling.

The correlation coefficient of two data sets is defined as^18^

2 $$r=\frac{\sum_{i=1}^{n}\left(x_{i}-\mathrm{x̅}\right)\left(y_{i}-\mathrm{y̅}\right)}{\left(n-1\right)s_{x}s_{y}}$$

where *x*_*i*_, *y*_*i*_ are values obtained at different temperatures, *x̅*, *y̅* are mean values, *s*_*x*_, *s*_*y*_ are standard deviations, and *n* is the number of (*x*_*i*_,*y*_*i*_) pairs. For identical data sets, *r* = 1. Lower *r* values are expected for IR spectra collected in two different phases (e.g., liquid and crystal) than those collected within the same phase. The matrix of the correlation coefficients of the IR spectra of 11OS5 is presented in Figure 6a. The data can be divided into four ranges: {373, 369, 365, 357 K}, Iso and N; {353, 349, 345, 341, 337, 333, 329 K}, SmA and SmC; {325, 321, 317, 313, 309, 305 K}, SmX; 301 K, crystallization; {297, 293, 289, 285, 281, 277, 273 K}, crystal. The correlation coefficients for some temperatures (e.g., 317 K) enable distinction of the N phase at 357 K (Figure 6b). The SmA → SmC transition is not shown in the correlation coefficient matrix.

**Figure 6:**
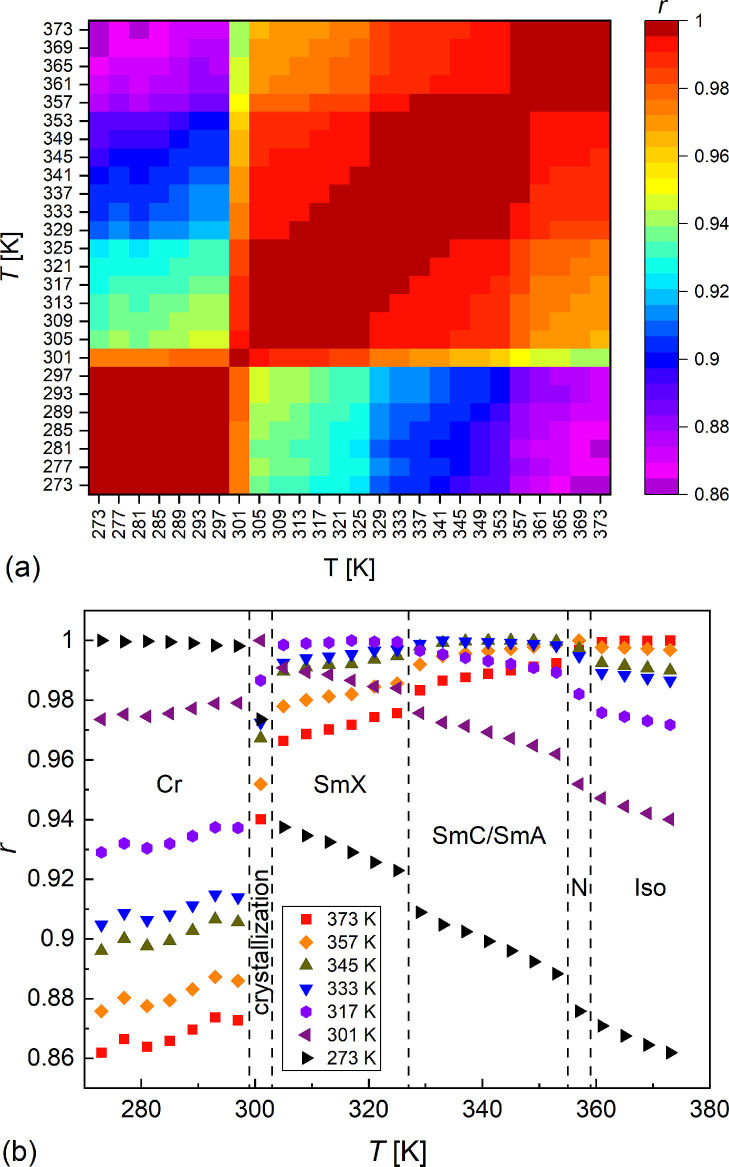
Correlation coefficient matrix of IR spectra of 11OS5 (a) and correlation coefficients for selected spectra as a function of temperature (b).

The k-means cluster analysis is based on an algorithm which separates data into clusters with maximal similarity of data within each cluster and maximal difference of data between clusters.^19–21^ The optimal number of clusters indicated by the “elbow method”^20^ is four (Figure S2 in Supporting Information). The true number of clusters in the data set is unknown. The elbow method is a heuristic technique used to estimate the optimal number of clusters by identifying the point where increasing the number of clusters no longer significantly reduces the within-cluster variance. The 4-cluster analysis performed for IR spectra in the whole range divides data as follows: {373, 369, 365, 357 K} – Iso and N; {353, 349, 345, 341, 337, 333, 329, 325, 321 K} – SmA, SmC, and SmX; {317, 313, 309, 305, 301 K} – SmX and crystallization; {297, 293, 289, 285, 281, 277, 273 K} – crystal. Thus, the 4-cluster analysis for full spectra is sensitive to the N → SmA and SmX → Cr transitions, but it does not correctly separate the temperature ranges of SmC and SmX. The Iso and N phases are not distinguished, the same as SmA and SmC. In the next step, the 4-cluster analysis was performed separately for selected spectral ranges to find out which intramolecular vibrations are sensitive to particular phase transitions, as described in Table 2.

**Table 2:** Band Assignment of the Experimental IR Spectra of 11OS5 at Room Temperature, Based on the def2TZVPP/B3LYP-D3­(BJ) Calculations in Gaussian^24^ and Potential Energy Distribution Analysis in VEDA^34,35^ (see Table S1 for Details).^a^

spectral range [cm^–1^]	main contributions
450–590	γPh_alkyl_, γPh_alkoxy_, βPh_alkoxy_, δCSC, δCCC_alkoxy_, δCOC_alkoxy_
590–685	γPh_alkoxy_, βPh_alkyl_, βPh_alkoxy_, γCOS
685–760	γPh_alkyl_, ρCH_2alkyl_, ρCH_2alkoxy_
760–860	γPh_alkyl_, γPh_alkoxy_, βPh_alkoxy_, ρCH_2alkoxy_, τCH_2alkyl_
860–930	βPh_alkoxy_, νCS
930–1065	νCC_alkoxy_, νCO_alkoxy_
1065–1190	βPh_alkyl_, βPh_alkoxy_, νCC_alkoxy_
1190–1290	βPh_alkoxy_, ωCH_2alkyl_
1290–1345	βPh_alkyl_, βPh_alkoxy_, ωCH_2alkyl_
1345–1426	βPh_alkyl_, ωCH_2alkyl_, ωCH_2alkoxy_
1426–1482	δCH_2alkyl_, δCH_2alkoxy_
1482–1550	βPh_alkyl_, βPh_alkoxy_, δCH_2alkoxy_
1550–1625	βPh_alkoxy_
1625–1750	νC=O
2700–3150	νCH

a Notations: β – in-plane deformation, γ – out-of-plane deformation, δ – scissoring, ν – stretching, ρ – rocking, τ – twisting, ω – wagging, Ph – phenyl ring.

The results of four-cluster analysis identical with those of full IR spectra are obtained for two spectral ranges (Figure 7): 590–685 cm^–1^ (deformations of aromatic rings and out-of-plane deformation of COS) and 1426–1482 cm^–1^ (scissoring of CH_2_ groups). The absorption bands sensitive to the Iso → N transition in the 4-cluster analysis are those in the 2700–3150 cm^–1^ range (stretching of C–H bonds). The N → SmA transition is indicated by bands at 590–685 cm^–1^, 760–860 cm^–1^, 1065–1190 cm^–1^, and 1290–1750 cm^–1^ (deformations of aromatic rings, out-of-plane deformation of the COS group, rocking, wagging, twisting, and scissoring of CH_2_ groups, and stretching of C–C and C=O bonds). The SmA and SmC phases are separated correctly for bands at 685–760 cm^–1^ (out-of-plane deformations of the aromatic ring next to the alkyl chain, rocking of the CH_2_ groups). The SmC → SmX transition is indicated by bands at 860–930 cm^–1^, 1065–1290 cm^–1^, and 1482–1625 cm^–1^ (deformations of aromatic rings, stretching of C–S in the COS group, stretching of C–C bonds, wagging and scissoring of CH_2_ groups). The crystallization is detected by all bands. However, the analysis for bands at 450–590 cm^–1^, 685–760 cm^–1^, and 930–1065 cm^–1^ (deformations of aromatic rings, scissoring of C–S–C, C–C–C, C–O–C, stretching of C–C, C–O bonds, rocking of CH_2_ groups) separates the spectrum collected at 273 K from other spectra collected in the 277–297 K range, which may indicate some structural changes within a solid state.

**Figure 7:**
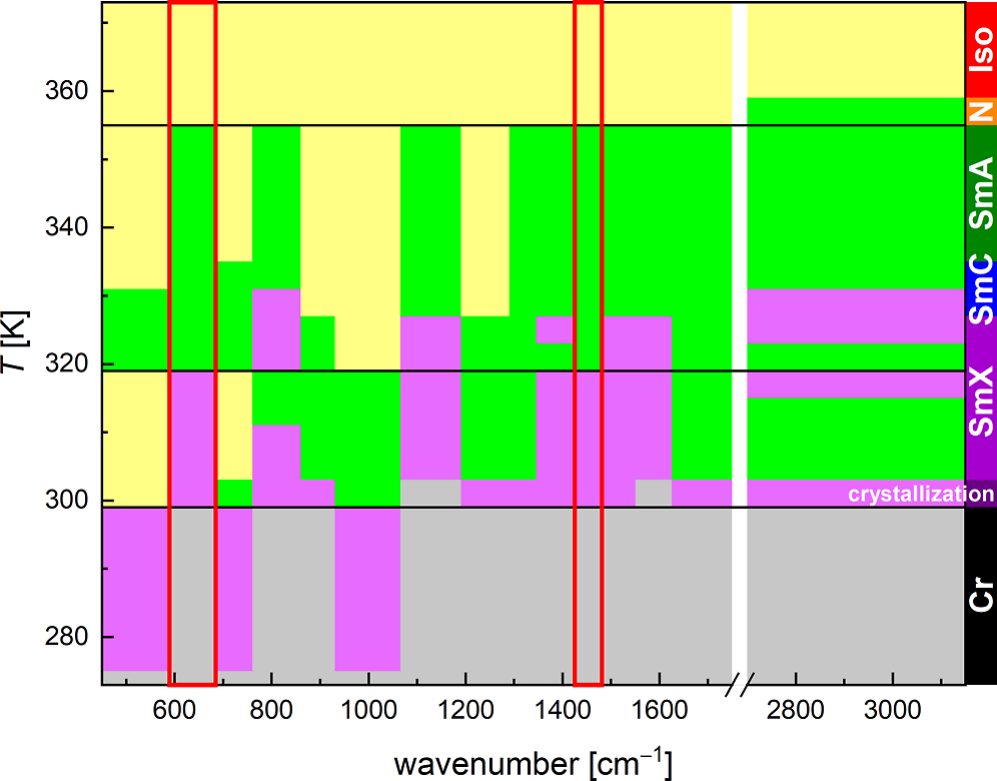
Results of the 4-cluster analysis for selected ranges in the IR spectra of 11OS5. Colors yellow, green, pink, and gray indicate separate clusters as a function of decreasing temperature. In some cases, spectra from two temperature ranges are assigned to one cluster. Horizontal lines indicate borders between clusters obtained for full IR spectra. Red frames indicate spectral ranges with identical clusters as obtained for full spectra.

Finally, the temperature behavior of the two selected absorption bands is investigated (Figure 8). The first band is located at 1663–1674 cm^–1^, corresponding to the C=O stretching (νC=O). The second band is located at 901–909 cm^–1^, corresponding to the in-plane deformations of the aromatic ring close to the alkoxy chain (β_asym_Ph_alkoxy_) and C–S stretching in the COS group (νCS). Each band is well separated from the neighboring absorption bands. The band related to νC=O shows a red shift (decrease of the band position) at transitions from the less ordered to more ordered phases on cooling, which is explained by formation of weak hydrogen bonds.^44,45^ The largest red shift of almost 8 cm^–1^ occurs between the SmX and crystal phases. The red shift at the SmC → SmX transition is very small, less than 1 cm^–1^, and comparable to the red shift observed at the Iso → N → SmA transitions. No red shift occurs at the SmA → SmC transition. The band related to β_asym_Ph_alkoxy_ and νCS vibrations shows the opposite behavior, which is a blue shift (increase of the band position) on cooling. The blue shift at the SmX → Cr and SmC → SmX transitions is 4 cm^–1^ and 1 cm^–1^, respectively, while it is negligible at transitions at higher temperatures.

**Figure 8:**
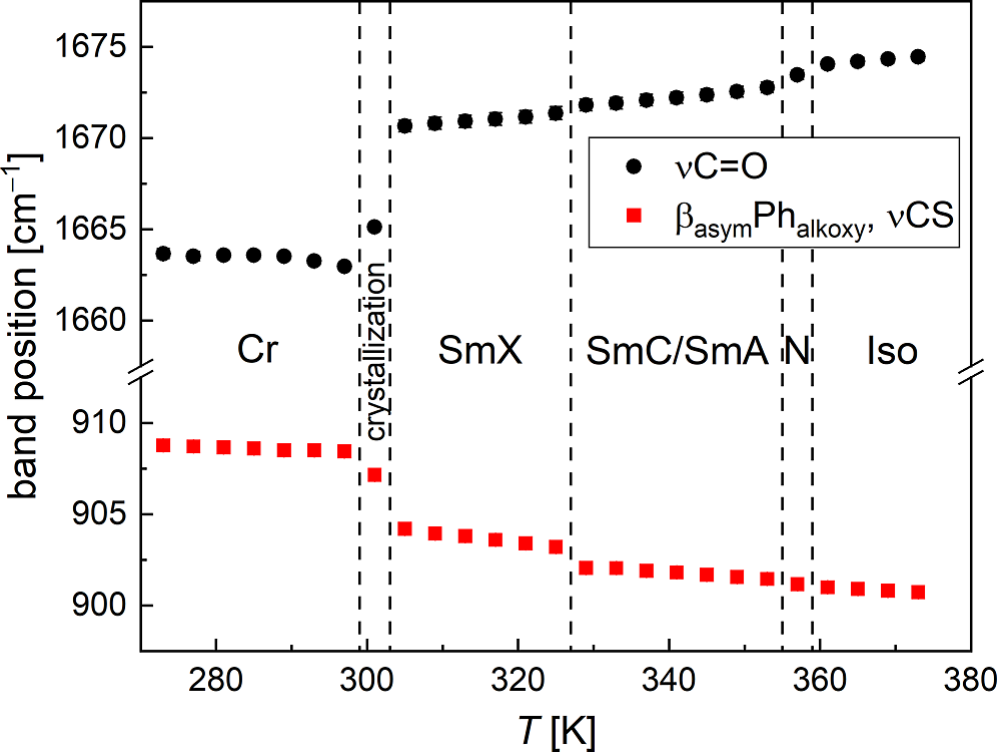
Positions of absorption bands related to νC=O and β_asym_Ph_alkoxy_, νCS vibrations in the 11OS5 molecule vs temperature.

The influence of intermolecular interactions on IR spectra was modeled by DFT calculations for dimers. The head-to-head and head-to-tail dimers of 11OS5 were optimized (Figure 9) and corresponding IR spectra were calculated using the 631+Gd basis set and B3LYP-D3­(BJ) functional (Figure 10), which gave a reasonably low RMSE and short computational time in calculations for an isolated molecule (Table 1). The theoretical band position at the 631+Gd/B3LYP-D3­(BJ) level related to νC=O is 1746 cm^–1^ for an isolated molecule (Table S7 in SI), 1741 and 1756 cm^–1^ for a head-to-head dimer, and 1726 and 1740 cm^–1^ for a head-to-tail dimer. The theoretical band position at the 631+Gd/B3LYP-D3­(BJ) level related to β_asym_Ph_alkoxy_ and νCS is 919 cm^–1^ for an isolated molecule (Table S7 in Supporting Information), 912 and 922 cm^–1^ for a head-to-head dimer, and 915 and 920 cm^–1^ for a head-to-tail dimer. The blue shift of the νC=O band (1756 cm^–1^) occurs when the C=O group is located close to the S atom from a neighbor molecule (C...S distance 4.0 Å) in a head-to-head dimer. The red shift (1726 cm^–1^) occurs when the C=O group interacts with the CH_2_ groups in the alkoxy chain from a neighbor molecule (O...H distance 2.6 Å) in a head-to-tail dimer. The latter result matches the experimental observations qualitatively. The band related to β_asym_Ph_alkoxy_ and νCS vibrations of the same molecule in a head-to-tail dimer is the weakly blue-shifted one at 920 cm^–1^, which also agrees qualitatively with experimental results. Thus, 11OS5 molecules in a crystal phase are likely in a head-to-tail configuration where C=O groups interact with alkoxy chains. There may also be an intercalated configuration, where C=O groups interact with alkyl chains. The head-to-head configuration is less likely, as it leads to proximity of C=O groups and S atoms from neighbor molecules, which would cause a blue shift of the νC=O band, against the experimental results. Moreover, the calculated energy of the head-to-tail dimer is lower by 2.2 kJ/mol than that of the head-to-head dimer.

**Figure 9:**
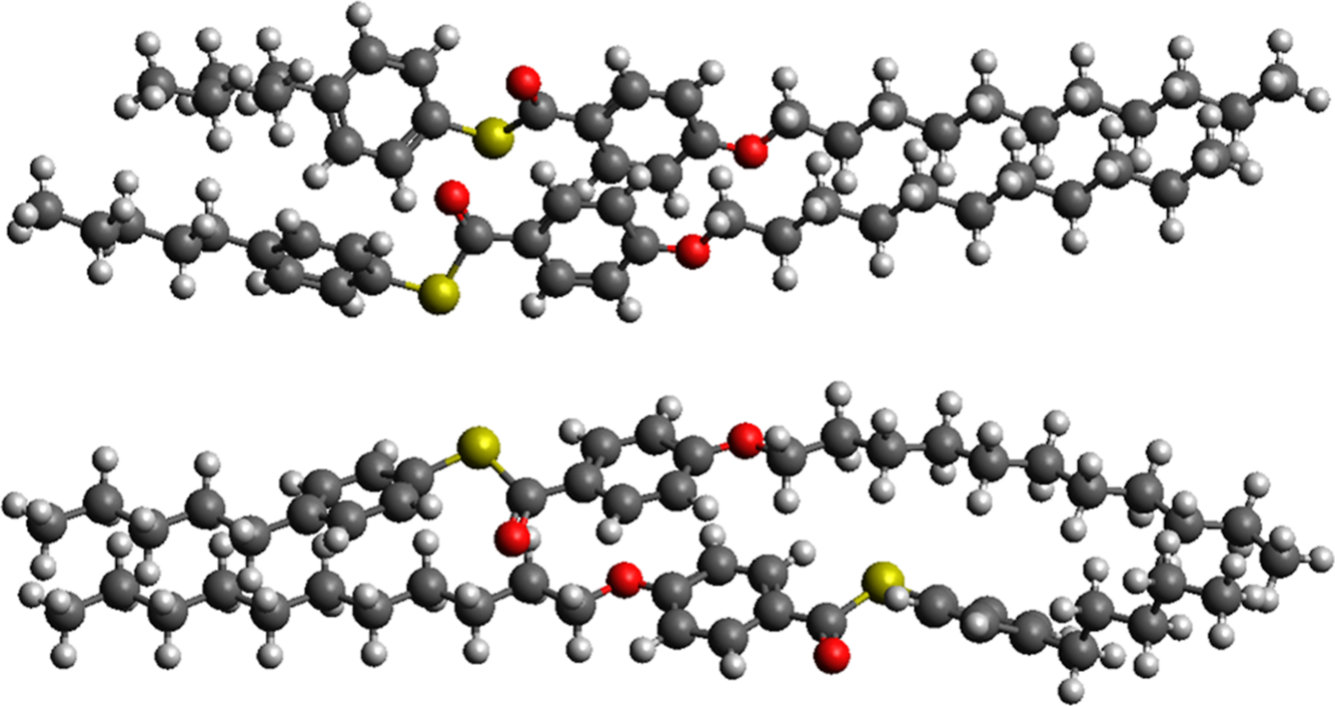
Head-to-head (top) and head-to-tail (bottom) dimers of 11OS5 optimized at the 631+Gd/B3LYP-D3­(BJ) level.

**Figure 10:**
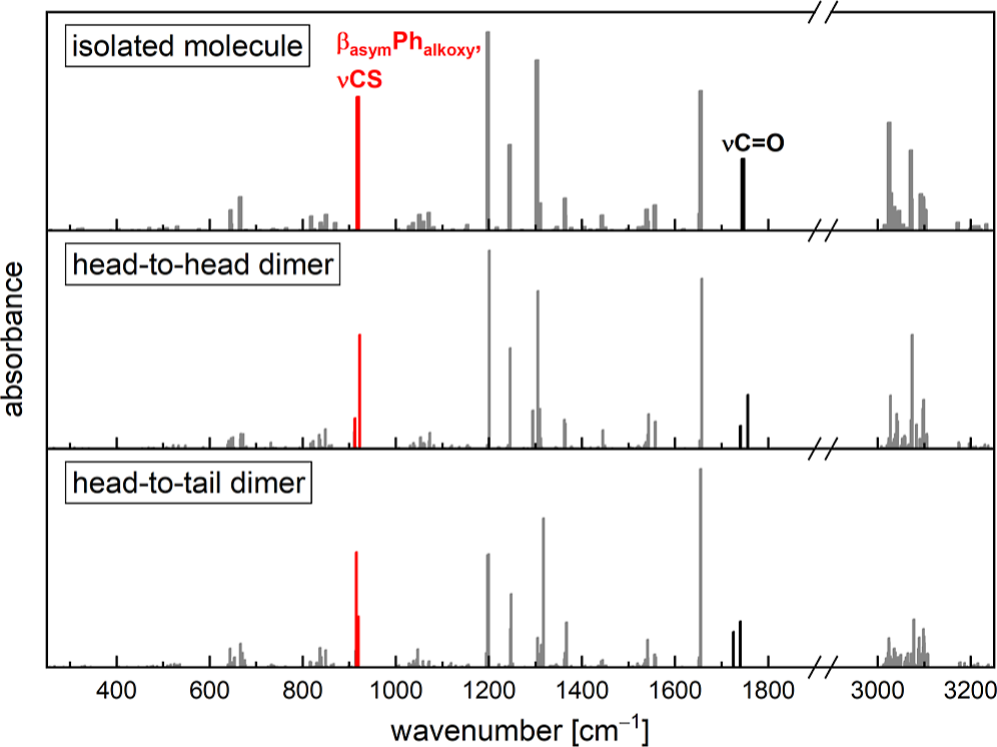
Theoretical unscaled IR spectra calculated for an isolated molecule, head-to-head dimer, and head-to-tail dimer of 11OS5 at the 631+Gd/B3LYP-D3­(BJ) level.

## 4 Summary and Conclusions

The isotropic liquid, nematic, smectic A, C, X, and crystal phases of the 11OS5 compound were investigated by dielectric and IR spectroscopies. The dielectric dispersion at 1 kHz is sensitive to all phase transitions, including the SmC → SmA transition. The dielectric spectra reveal two relaxation processes in the Iso, N, SmA, and SmC phases: low-frequency Maxwell–Wagner–Sillars relaxation and a high-frequency molecular s-process, related to rotations around the short molecular axes (flip-flop motion). The MWS process is out of the frequency range in the hexagonal SmX phase. The s-process is also strongly shifted to lower frequencies at the SmC → SmX transition, by more than 1 order of magnitude. The activation energy of the s-process increases to 144 kJ/mol in SmX compared to 94 kJ/mol in SmA and SmC. No relaxation processes are detected in the crystal phase.

The IR absorption bands were assigned to intramolecular vibrations based on DFT calculations performed with def2TZVPP, def2TZVP, def2SVPP, def2SVP, 6311+Gdp, and 631+Gd basis sets and B3LYP-D3­(BJ) or BLYP-D3­(BJ) exchange–correlation functionals. The def2TZVPP/B3LYP-D3­(BJ) level of theory provides the lowest root-mean square error between experimental and scaled calculated band positions (11.6 cm^–1^). However, def2TZVP/B3LYP-D3­(BJ) gives a negligibly higher RMSE (11.9 cm^–1^) with twice as short calculations as those for def2TZVPP/B3LYP-D3­(BJ). The third best performance is obtained for 631+Gd/B3LYP-D3­(BJ), which provides RMSE = 12.5 cm^–1^, while computational time is ten times shorter than that for def2TZVPP/B3LYP-D3­(BJ). The 631+Gd/B3LYP-D3­(BJ) level was further applied for calculations of the theoretical spectra of 11OS5 dimers in head-to-head and head-to-tail configurations.

The IR spectra collected as a function of temperature were analyzed by calculation of the correlation coefficient matrix, which was sensitive to most of the phase transitions except the SmC → SmA transition. The k-means cluster analysis performed for the full spectra was sensitive to the N → SmA and SmX → Cr transition, while the Iso → N, SmA → SmC transitions were not detected, and the SmC → SmX transition temperature was underestimated. The k-means cluster analysis performed separately in various spectral ranges showed that different intramolecular vibrations had different sensitivities to particular phase transitions. In some cases, IR spectra collected in the same phase were placed in separate clusters by the algorithm. Thus, prior knowledge of the phase sequence is necessary, and the k-means cluster analysis should be used mainly to indicate which absorption bands are the most sensitive to the phase transition in question, at least in a situation where the ordering of molecules occurs gradually on cooling, as it is for liquid crystals.

The absorption band at 1663–1674 cm^–1^, related to the C=O stretching, and the band at 901–909 cm^–1^, related to the in-plane deformations of the aromatic ring close to the alkoxy chain and C–S stretching in the COS group, show a red shift and a blue shift with decreasing temperature, respectively. The Iso → N and N → SmA transitions influence mainly the band at 1663–1674 cm^–1^ (red shift of ∼1 cm^–1^ at each transition). The IR spectra of SmC and SmA cannot be distinguished by any applied analytical method because the molecular arrangement and, consequently, intermolecular interactions in these phases are very similar, as the tilt angle in SmC is only 11° and the smectic layer shrinkage between SmA and SmC is ∼1%.^16^ The red shift/blue shift of the former/latter band at the SmC → SmX transition is only ∼1 cm^–1^, despite the fact that the SmX phase is characterized by the long-range positional order in three dimensions. It is caused by the preserved orientational disorder in the SmX phase, detected in dielectric spectra as the s-process. The strongest red shift/blue shift (8/4 cm^–1^) of the former/latter band occurs during crystallization. It agrees with the absence of dielectric relaxation processes in the solid state, indicating the lack of orientational and conformational disorder in a crystal phase of 11OS5. The DFT calculations for dimers indicate that the C=O group interacts rather with the terminal chains than with the S atom; therefore, the head-to-tail or intercalated configuration in a crystal phase is more likely than the head-to-head configuration.

The main conclusions from both experimental methods are (1) transitions between less-ordered smectic phases often lead to only small differences in molecular motions and intramolecular interactions, and more than one experimental or analytical method is necessary to detect them; (2) the rise of the hexagonal order within smectic layers slows down significantly the s-process, while the intramolecular vibrations are weakly affected; (3) comparison of vibrational spectra in the crystal and liquid/liquid crystal phases, together with theoretical calculations of intermolecular interactions, can give some insight into the molecular arrangement in the crystal phase, even when its exact structure is unknown.

## Supplementary Material



## Notes

Tables S1–S8 – experimental and calculated (scaled and unscaled) positions of IR absorption bands at different levels of theory together with band assignments and % contributions of different vibrational modes; Figure S1 – experimental vs unscaled calculated positions of IR absorption bands with linear fits for determination of scaling coefficients; and Figure S2 – determination of an optimal number of clusters in the k-means cluster analysis by the “elbow method” (PDF)
