# Supplementary material for: Transitions between Liquid Crystalline Phases Investigated by Dielectric and Infrared Spectroscopies
Source: J Phys Chem B. 2026 Jan 28;130(6):1974–83. doi: 10.1021/acs.jpcb.5c07310 (PMC13296728; doi:10.1021/acs.jpcb.5c07310)
Supplement: Supplementary file 1 [file jp5c07310_si_001.pdf]

Transitions between Liquid Crystalline Phases Investigated by Dielectric and Infra-Red Spectroscopies

Aleksandra Deptuch [a,*], Natalia Osiecka-Drewniak [a], Anna Paliga [b], Natalia Górska [c], Anna Drzewicz [a], Katarzyna Chat [a], Mirosława D. Ossowska-Chruściel [d], Janusz Chruściel [d]

[a] Institute of Nuclear Physics Polish Academy of Sciences, Radzikowskiego 152, PL-31342 Kraków, Poland

[b] Faculty of Physics, Astronomy and Applied Computer Science, Jagiellonian University, Łojasiewicza 11, PL-30348 Kraków, Poland

[c] Faculty of Chemistry, Jagiellonian University, Gronostajowa 2, PL-30387 Kraków, Poland

[d] Faculty of Science, University of Siedlce, 3 Maja 54, PL-08110 Siedlce, Poland

* corresponding author, aleksandra.deptuch@ifj.edu.pl

Supporting Information

Table S1. The band assignment of the experimental IR spectra of 11OS5 at the room temperature, based on the DFT calculations (**def2TZVPP** basis set, **B3LYP-D3(BJ)** functional). Notations: β – in-plane deformation, γ – out-of-plane deformation, δ – scissoring, ν – stretching, ρ – rocking, τ – twisting, ω – wagging.

experimental peak position [cm^{-1}]	unscaled calculated peak position [cm^{-1}]	scaled calculated peak position [cm^{-1}]	vibration (contribution ≥ 10%)
384	387	377	58% δCCC_{alkyl}
401	403	393	27% δCCC_{alkoxy}
416	417	406	83% γPh_{alkyl}
445	448	436	20% δCCO_{alkoxy}
468	472	460	40% δCCC_{alkoxy}
484	495	482	12% γPh_{alkyl}
500	505	492	40% δCCC_{alkyl}
506	515	502	73% γPh_{alkoxy}
523	532	518	49% δCOC_{alkoxy}
570	579	564	30% $\beta_{asym}Ph_{alkoxy}$, 10% δCOC_{alkoxy}, 10% $\delta CSC/Ph_{alkyl}$
628	648	631	65% $\beta_{asym}Ph_{alkoxy}$
641	657	640	51% γCOS, 16% γPh_{alkoxy}
661	669	652	44% $\beta_{asym}Ph_{alkoxy}$
670	680	662	58% $\beta_{sym}Ph_{alkyl}$
723	734	715	83% $\rho CH_{2alkoxy}$
733	742	723	71% ρCH_{2alkyl}
750	766	746	22% ρCH_{2alkyl}, 17% γPh_{alkyl}
781	792	772	45% $\rho CH_{2alkoxy}$
803	822	801	51% γPh_{alkyl}
819	841	819	47% $\beta_{asym}Ph_{alkoxy}$
839	863	841	76% γPh_{alkoxy}
851	871	848	49% γPh_{alkyl}, 20% τCH_{2alkyl}
906	919	895	29% $\beta_{asym}Ph_{alkoxy}$, 26% νCS
934	993	967	74% νCC_{alkoxy}
946	1001	975	57% νCC_{alkoxy}
987	1028	1001	69% νCC_{alkoxy}
1015	1048	1021	41% νCO_{alkoxy}
1023	1066	1038	59% νCC_{alkoxy}
1051	1076	1048	67% νCC_{alkoxy}
1095	1120	1091	75% $\beta_{asym}Ph_{alkyl}$
1119	1150	1120	54% $\nu_{sym}CCC_{alkoxy}$
1168	1189	1158	73% $\beta_{sym}Ph_{alkoxy}$
1212	1238	1206	69% $\beta_{asym}Ph_{alkoxy}$
1231	1266	1233	59% ωCH_{2alkyl}
1269	1296	1262	66% $\beta_{asym}Ph_{alkoxy}$
1310	1348	1313	69% $\beta_{asym}Ph_{alkoxy}$
1323	1355	1320	40% ωCH_{2alkyl}, 25% $\beta_{asym}Ph_{alkyl}$
1353	1370	1335	61% $\omega CH_{2alkoxy}$
1378	1395	1359	55% ωCH_{2alkyl}
1400	1432	1395	65% $\omega CH_{2alkoxy}$
1421	1444	1407	53% $\beta_{asym}Ph_{alkyl}$
1435	1477	1439	81% δCH_{2alkyl}
1468	1523	1484	81% $\delta CH_{2alkoxy}$
1495	1535	1495	67% $\beta_{asym}Ph_{alkyl}$
1508	1549	1509	63% $\beta_{asym}Ph_{alkoxy}$
1600	1646	1603	70% $\beta_{sym}Ph_{alkoxy}$
1661	1736	1691	89% $\nu C{=}O$
2849	3017	2871	74% $\nu_{sym}CH_{2alkoxy}$
2872	3034	2887	93% $\nu_{sym}CH_{2alkoxy}$
2915	3060	2912	83% $\nu_{asym}CH_{2alkoxy}$
2956	3078	2929	75% $\nu_{asym}CH_{2alkoxy}$
3041	3190	3035	97% $\nu_{asym}CH/Ph_{alkoxy}$
3049	3200	3045	88% $\nu_{sym}CH/Ph_{alkyl}$
3060	3221	3065	99% $\nu_{sym}CH/Ph_{alkoxy}$

Table S2. The band assignment of the experimental IR spectra of 11OS5 at the room temperature, based on the DFT calculations (**def2TZVP** basis set, **B3LYP-D3(BJ)** functional). Notations: β – in-plane deformation, γ – out-of-plane deformation, δ – scissoring, ν – stretching, ρ – rocking, τ – twisting, ω – wagging.

experimental peak position [cm^{-1}]	unscaled calculated peak position [cm^{-1}]	scaled calculated peak position [cm^{-1}]	vibration (contribution ≥ 10%)
384	388	378	59% δCCC_{alkyl}
401	403	393	25% δCCC_{alkoxy}
416	415	404	80% γPh_{alkyl}
445	448	437	30% δCCC_{alkoxy}
468	472	460	46% δCCO_{alkoxy}
484	495	482	13% γPh_{alkyl}, 11% δCCO_{alkoxy}
500	505	492	44% δCCC_{alkyl}
506	514	502	67% γPh_{alkoxy}
523	532	519	45% δCOC_{alkoxy}
570	579	564	25% $\beta_{asym}Ph_{alkoxy}$, 12% δCOC_{alkoxy}
628	647	631	66% $\beta_{asym}Ph_{alkoxy}$
641	657	640	77% γPh_{alkoxy}
661	669	652	51% $\beta_{asym}Ph_{alkoxy}$
670	680	663	45% $\beta_{sym}Ph_{alkyl}$
723	735	715	68% $\rho CH_{2alkoxy}$
733	742	723	67% ρCH_{2alkyl}
750	766	747	36% ρCH_{2alkyl}
781	792	772	46% $\rho CH_{2alkoxy}$
803	820	799	56% γPh_{alkyl}
819	841	820	60% $\beta_{asym}Ph_{alkoxy}$
839	860	838	82% γPh_{alkoxy}
851	869	847	48% γPh_{alkyl}, 10% ρCH_{2alkyl}
906	919	896	29% $\beta_{asym}Ph_{alkoxy}$, 25% νCS
934	993	968	66% νCC_{alkoxy}
946	1001	976	69% νCC_{alkoxy}
987	1028	1002	57% νCC_{alkoxy}
1015	1048	1021	51% νCC_{alkoxy}
1023	1067	1040	73% νCC_{alkoxy}
1051	1077	1050	66% νCC_{alkoxy}
1095	1120	1091	69% $\beta_{asym}Ph_{alkyl}$
1119	1150	1121	35% $\nu_{sym}CCC_{alkoxy}$, 12% $\omega CH_{2alkoxy}$
1168	1188	1158	61% $\beta_{sym}Ph_{alkoxy}$
1212	1238	1206	70% $\beta_{asym}Ph_{alkoxy}$
1231	1265	1233	56% ωCH_{2alkyl}
1269	1295	1262	49% νCO_{alkoxy}, 11% $\beta_{asym}Ph_{alkoxy}$
1310	1348	1314	61% $\beta_{asym}Ph_{alkoxy}$
1323	1354	1319	48% ωCH_{2alkyl}, 25% $\beta_{asym}Ph_{alkyl}$
1353	1369	1334	58% $\omega CH_{2alkoxy}$
1378	1395	1359	53% ωCH_{2alkyl}
1400	1430	1393	70% $\omega CH_{2alkoxy}$
1421	1443	1406	57% $\beta_{asym}Ph_{alkyl}$, 11% ωCH_{2alkyl}
1435	1476	1438	69% δCH_{2alkyl}
1468	1522	1483	69% $\delta CH_{2alkoxy}$
1495	1534	1495	58% $\beta_{asym}Ph_{alkyl}$
1508	1548	1508	50% $\beta_{asym}Ph_{alkoxy}$, 12% νCO_{alkoxy}
1600	1646	1604	66% $\beta_{sym}Ph_{alkoxy}$
1661	1736	1692	89% $\nu C{=}O$
2849	3017	2871	85% $\nu_{sym}CH_{2alkoxy}$
2872	3034	2887	92% $\nu_{sym}CH_{2alkoxy}$
2915	3060	2912	90% $\nu_{asym}CH_{2alkoxy}$
2956	3077	2928	90% $\nu_{asym}CH_{2alkoxy}$
3041	3190	3036	98% $\nu_{asym}CH/Ph_{alkoxy}$
3049	3199	3044	88% $\nu_{sym}CH/Ph_{alkyl}$
3060	3220	3064	85% $\nu_{sym}CH/Ph_{alkoxy}$, 15% $\nu_{sym}CH_{2alkoxy}$

Table S3. The band assignment of the experimental IR spectra of 11OS5 at the room temperature, based on the DFT calculations (**def2SVPP** basis set, **B3LYP-D3(BJ)** functional). Notations: β – in-plane deformation, γ – out-of-plane deformation, δ – scissoring, ν – stretching, ρ – rocking, τ – twisting, ω – wagging.

experimental peak position [cm^{-1}]	unscaled calculated peak position [cm^{-1}]	scaled calculated peak position [cm^{-1}]	vibration (contribution ≥ 10%)
384	389	376	65% δCCC_{alkyl}
401	406	392	16% δCCC_{alkoxy}
416	421	407	70% γPh_{alkyl}
445	452	436	17% δCCO_{alkoxy}
468	476	460	54% δCCC_{alkoxy}, 10% νCS
484	500	483	11% δCCC_{alkyl}
500	507	490	41% δCCC_{alkyl}
506	521	503	72% γPh_{alkoxy}
523	535	517	41% δCCC_{alkoxy}
570	582	562	16% $\beta_{asym}Ph_{alkoxy}$, 11% $\delta CSC/Ph_{alkyl}$
628	647	625	77% $\beta_{asym}Ph_{alkoxy}$
641	656	633	83% γPh_{alkoxy}
661	671	648	26% $\beta_{asym}Ph_{alkoxy}$
670	680	657	29% $\beta_{sym}Ph_{alkyl}$, 21% $\nu SC/Ph_{alkyl}$
723	744	718	77% $\rho CH_{2alkoxy}$
733	751	725	76% ρCH_{2alkyl}
750	772	745	15% γPh_{alkyl}, 11% ρCH_{2alkyl}
781	798	771	49% $\rho CH_{2alkoxy}$
803	824	796	44% γPh_{alkyl}, 10% ρCH_{2alkyl}
819	846	817	56% $\beta_{asym}Ph_{alkoxy}$
839	868	838	75% γPh_{alkoxy}
851	873	843	34% γPh_{alkyl}, 17% ρCH_{2alkyl}
906	928	896	37% νCS, 23% $\beta_{asym}Ph_{alkoxy}$
934	1014	979	68% νCC_{alkoxy}
946	1026	991	74% $\beta_{asym}Ph_{alkoxy}$
987	1035	999	65% $\beta_{asym}Ph_{alkyl}$
1015	1084	1041	68% νCC_{alkoxy}
1023	1090	1047	69% νCC_{alkoxy}
1051	1118	1073	40% $\nu_{sym}CCC_{alkoxy}$
1095	1136	1091	89% $\beta_{sym}Ph_{alkoxy}$
1119	1154	1108	47% $\nu_{sym}CCC_{alkoxy}$
1168	1181	1134	87% $\beta_{sym}Ph_{alkoxy}$
1212	1248	1198	59% $\beta_{asym}Ph_{alkoxy}$
1231	1301	1249	60% $\omega CH_{2alkoxy}$
1269	1325	1272	59% $\beta_{asym}Ph_{alkoxy}$
1310	1377	1322	52% $\beta_{asym}Ph_{alkoxy}$
1323	1399	1343	58% $\omega CH_{2alkoxy}$
1353	1401	1345	60% ωCH_{2alkyl}
1378	1419	1362	50% $\omega CH_{2alkoxy}$
1400	1446	1388	46% $\omega CH_{2alkoxy}$
1421	1449	1391	52% $\beta_{asym}Ph_{alkyl}$, 11% ωCH_{2alkyl}
1435	1482	1423	98% δCH_{3alkyl}
1468	1512	1452	74% $\delta CH_{2alkoxy}$
1495	1538	1477	76% $\beta_{asym}Ph_{alkyl}$
1508	1558	1496	81% $\beta_{asym}Ph_{alkoxy}$
1600	1671	1604	72% $\beta_{sym}Ph_{alkoxy}$
1661	1787	1716	91% νC=O
2849	3006	2862	82% $\nu_{sym}CH_{2alkoxy}$
2872	3025	2880	96% $\nu_{sym}CH_{2alkoxy}$
2915	3060	2914	89% $\nu_{asym}CH_{2alkoxy}$
2956	3079	2932	80% $\nu_{asym}CH_{2alkoxy}$
3041	3191	3039	95% $\nu_{asym}CH/Ph_{alkoxy}$
3049	3203	3050	95% $\nu_{sym}CH/Ph_{alkyl}$
3060	3219	3065	90% $\nu_{sym}CH/Ph_{alkoxy}$

Table S4. The band assignment of the experimental IR spectra of 11OS5 at the room temperature, based on the DFT calculations (**def2SVP** basis set, **B3LYP-D3(BJ)** functional). Notations: β – in-plane deformation, γ – out-of-plane deformation, δ – scissoring, ν – stretching, ρ – rocking, τ – twisting, ω – wagging.

experimental peak position [cm^{-1}]	unscaled calculated peak position [cm^{-1}]	scaled calculated peak position [cm^{-1}]	vibration (contribution ≥ 10%)
384	388	376	57% δCCC_{alkyl}
401	406	393	44% δCCC_{alkoxy}
416	421	408	67% γPh_{alkyl}
445	451	437	20% δCCC_{alkoxy}
468	475	460	47% δCCC_{alkoxy}
484	499	483	21% γPh_{alkyl}
500	507	491	32% δCCC_{alkyl}, 18% γPh_{alkyl}
506	520	504	74% γPh_{alkoxy}
523	535	518	45% δCCO_{alkoxy}, 11% γPh_{alkyl}
570	581	563	11% $\beta_{asym}Ph_{alkoxy}$, 10% δCCO_{alkoxy}
628	646	626	47% $\beta_{asym}Ph_{alkoxy}$
641	655	634	60% γCOS, 14% γPh_{alkoxy}
661	671	650	37% $\beta_{asym}Ph_{alkoxy}$
670	680	659	60% $\beta_{sym}Ph_{alkyl}$
723	741	718	69% $\rho CH_{2alkoxy}$
733	745	722	69% ρCH_{2alkyl}
750	767	743	18% ρCH_{2alkyl}, 15% γPh_{alkyl}
781	790	765	61% $\rho CH_{2alkoxy}$
803	820	794	51% ρCH_{2alkyl}
819	846	819	57% $\beta_{asym}Ph_{alkoxy}$
839	866	839	62% γPh_{alkoxy}
851	867	840	35% γPh_{alkyl}, 11% γPh_{alkoxy}
906	928	899	40% νCS, 25% $\beta_{asym}Ph_{alkoxy}$
934	1012	980	75% νCC_{alkoxy}
946	1022	990	73% $\beta_{asym}Ph_{alkoxy}$
987	1032	1000	74% $\beta_{asym}Ph_{alkoxy}$
1015	1081	1043	55% νCC_{alkoxy}
1023	1088	1049	57% νCC_{alkoxy}
1051	1113	1073	36% νCC_{alkoxy}
1095	1132	1092	89% $\beta_{asym}Ph_{alkoxy}$
1119	1150	1109	72% $\nu_{sym}CCC_{alkoxy}$
1168	1177	1135	88% $\beta_{sym}Ph_{alkoxy}$
1212	1246	1202	66% $\beta_{asym}Ph_{alkoxy}$
1231	1287	1241	60% $\omega CH_{2alkoxy}$
1269	1320	1273	26% $\omega CH_{2alkoxy}$, 21% $\beta_{asym}Ph_{alkoxy}$
	1326	1279	27% $\omega CH_{2alkoxy}$, 20% $\beta_{asym}Ph_{alkoxy}$
1310	1374	1325	58% $\beta_{asym}Ph_{alkoxy}$
1323	1386	1337	61% $\omega CH_{2alkoxy}$
1353	1389	1341	50% ωCH_{2alkyl}
1378	1407	1357	66% $\omega CH_{2alkoxy}$, 10% νCC_{alkoxy}
1400	1435	1384	51% $\omega CH_{2alkoxy}$
1421	1443	1392	52% $\beta_{asym}Ph_{alkyl}$, 15% ωCH_{2alkyl}
1435	1471	1419	98% δCH_{3alkyl}
1468	1500	1447	60% $\delta CH_{2alkoxy}$
1495	1530	1476	80% $\beta_{asym}Ph_{alkyl}$
1508	1552	1497	80% $\beta_{asym}Ph_{alkoxy}$
1600	1667	1608	75% $\beta_{sym}Ph_{alkoxy}$
1661	1787	1724	90% νC=O
2849	3018	2866	87% $\nu_{sym}CH_{2alkoxy}$
2872	3038	2885	96% $\nu_{sym}CH_{2alkoxy}$
2915	3071	2916	91% $\nu_{asym}CH_{2alkoxy}$
2956	3090	2934	84% $\nu_{asym}CH_{2alkoxy}$
3041	3196	3035	95% $\nu_{asym}CH/Ph_{alkoxy}$
3049	3207	3045	97% $\nu_{sym}CH/Ph_{alkyl}$
3060	3224	3061	99% $\nu_{sym}CH/Ph_{alkoxy}$

Table S5. The band assignment of the experimental IR spectra of 11OS5 at the room temperature, based on the DFT calculations (**def2SVP** basis set, **BLYP-D3(BJ)** functional). Notations: β – in-plane deformation, γ – out-of-plane deformation, δ – scissoring, ν – stretching, ρ – rocking, τ – twisting, ω – wagging.

experimental peak position [cm^{-1}]	unscaled calculated peak position [cm^{-1}]	scaled calculated peak position [cm^{-1}]	vibration (contribution ≥ 10%)
384	378	380	61% δCCC_{alkyl}
401	392	394	34% δCCC_{alkoxy}, 10% νCS
416	423	425	33% $\nu_{asym}CSC/Ph_{alkyl}$, 10% δCCC_{alkyl}
445	438	440	12% δCCO_{alkoxy}
468	460	462	47% $\delta COC/Ph_{alkoxy}$
484	485	487	11% δCOC_{alkoxy}
500	493	495	40% δCCC_{alkyl}
506	504	506	30% δCCC_{alkoxy}
523	519	522	49% δCOC_{alkoxy}
570	560	563	23% $\delta CSC/Ph_{alkyl}$, 20% δCCC_{alkyl}, 12% δCOC_{alkoxy}
628	627	630	58% $\beta_{asym}Ph_{alkoxy}$, 11% γCOS
641	629	632	52% γCOS, 12% γPh_{alkoxy}
661	647	650	42% $\beta_{asym}Ph_{alkoxy}$
670	657	660	31% $\nu SC/Ph_{alkyl}$, 18% $\beta_{sym}Ph_{alkyl}$
723	730	734	75% ρCH_{2alkyl}
733	737	741	74% γPh_{alkoxy}
750	746	750	36% ρCH_{2alkyl}, 27% γPh_{alkyl}
781	772	776	37% $\rho CH_{2alkoxy}$
803	796	800	58% ρCH_{2alkyl}
819	814	818	34% $\beta_{asym}Ph_{alkoxy}$
839	837	841	78% γPh_{alkoxy}
851	842	846	40% ρCH_{2alkyl}, 11% γPh_{alkyl}
906	888	892	36% νCS, 33% $\beta_{asym}Ph_{alkoxy}$
934	956	961	88% γPh_{alkoxy}
946	962	967	92% γPh_{alkyl}
987	974	979	50% νCC_{alkoxy}
1015	1002	1005	49% νCC_{alkoxy}
1023	1013	1016	40% νCO_{alkoxy}
1051	1047	1051	51% νCC_{alkoxy}
1095	1078	1082	75% $\beta_{asym}Ph_{alkyl}$
1119	1102	1106	88% $\beta_{sym}Ph_{alkoxy}$
1168	1144	1148	73% $\beta_{sym}Ph_{alkoxy}$
1212	1202	1206	66% $\beta_{asym}Ph_{alkoxy}$
1231	1219	1223	53% ωCH_{2alkyl}
1269	1265	1269	55% νCO_{alkoxy}, 14% $\beta_{asym}Ph_{alkoxy}$
1310	1340	1345	65% $\beta_{sym}Ph_{alkoxy}$
1323	1345	1350	33% $\omega CH_{2alkoxy}$, 13% $\beta_{sym}Ph_{alkoxy}$
1353	1350	1355	57% ωCH_{2alkyl}, 14% $\beta_{sym}Ph_{alkyl}$
1378	1359	1364	67% $\omega CH_{2alkoxy}$
1400	1384	1389	52% $\omega CH_{2alkoxy}$
1421	1396	1401	60% $\beta_{asym}Ph_{alkyl}$, 13% ωCH_{2alkyl}
1435	1432	1437	86% δCH_{3alkyl}
1468	1457	1462	69% $\delta CH_{2alkoxy}$
1495	1478	1483	77% $\beta_{asym}Ph_{alkyl}$
1508	1494	1499	57% $\beta_{asym}Ph_{alkoxy}$
1600	1603	1608	67% $\beta_{sym}Ph_{alkoxy}$
1661	1707	1713	90% νC=O
2849	2939	2866	79% $\nu_{sym}CH_{2alkoxy}$
2872	2958	2885	96% $\nu_{sym}CH_{2alkoxy}$
2915	2988	2914	91% $\nu_{asym}CH_{2alkoxy}$
2956	3006	2932	83% $\nu_{asym}CH_{2alkoxy}$
3041	3113	3036	96% $\nu_{asym}CH/Ph_{alkoxy}$
3049	3124	3047	99% $\nu_{sym}CH/Ph_{alkyl}$
3060	3141	3063	100% $\nu_{sym}CH/Ph_{alkoxy}$

Table S6. The band assignment of the experimental IR spectra of 11OS5 at the room temperature, based on the DFT calculations (**6311+Gdp** basis set, **B3LYP-D3(BJ)** functional). Notations: β – in-plane deformation, γ – out-of-plane deformation, δ – scissoring, ν – stretching, ρ – rocking, τ – twisting, ω – wagging.

experimental peak position [cm^{-1}]	unscaled calculated peak position [cm^{-1}]	scaled calculated peak position [cm^{-1}]	vibration (contribution ≥ 10%)
384	381	373	35% δCCC_{alkoxy}, 13% $\delta OCC/Ph_{alkoxy}$
401	402	393	25% δCCC_{alkoxy}
416	422	413	28 % νSC, 25% δCCC_{alkyl}
445	447	437	17% δCCC_{alkoxy}
468	473	463	13% $\delta OCC/Ph_{alkoxy}$
484	507	496	32% δCCC_{alkoxy}
500	508	497	77% γPh_{alkoxy}
506	519	508	38% δCCC_{alkoxy}
523	546	534	17% γPh_{alkyl}
570	585	572	14% γPh_{alkyl}
628	645	631	74% $\beta_{asym} Ph_{alkoxy}$
641	646	632	80% γPh_{alkoxy}
661	665	650	40% $\beta_{asym} Ph_{alkoxy}$
670	673	658	30% $\beta_{sym} Ph_{alkyl}$
723	733	717	65% $\rho CH2_{alkyl}$
733	742	726	73% $\rho CH2_{alkoxy}$
750	757	740	75% γPh_{alkyl}
781	791	774	53% $\rho CH2_{alkoxy}$
803	824	806	49% γPh_{alkyl}
819	838	820	29% νCC_{alkoxy}, 17% νCO_{alkoxy}, 13% $\beta_{asym} Ph_{alkoxy}$
839	850	831	68% γPh_{alkoxy}
851	850	831	49% γPh_{alkyl}
906	914	894	24% νCS, 22% $\beta_{asym} Ph_{alkoxy}$
934	985	963	86% γPh_{alkoxy}
946	999	977	55% νCC_{alkoxy}
987	1025	1002	41% νCC_{alkoxy}, 21% $\beta_{asym} Ph_{alkoxy}$
1015	1044	1018	49% νCO_{alkoxy}
1023	1064	1037	70% νCC_{alkoxy}
1051	1075	1048	63% νCC_{alkoxy}
1095	1114	1086	78% $\beta_{asym} Ph_{alkyl}$
1119	1147	1118	58% $\nu_{sym} CCC_{alkoxy}$
1168	1186	1156	62% $\beta_{sym} Ph_{alkoxy}$
1212	1236	1205	71% $\beta_{asym} Ph_{alkoxy}$
1231	1266	1234	67% $\omega CH2_{alkoxy}$
1269	1290	1258	46% νCO_{alkoxy}, 23% $\beta_{asym} Ph_{alkoxy}$
1310	1347	1313	72% $\beta_{asym} Ph_{alkoxy}$
1323	1348	1314	52% $\beta_{asym} Ph_{alkyl}$, 14% $\tau CH2_{alkyl}$
1353	1365	1331	65% $\omega CH2_{alkoxy}$
1378	1413	1377	83% $\delta CH3_{alkoxy}$
1400	1430	1394	57% $\omega CH2_{alkoxy}$
1421	1435	1399	37% $\tau CH2_{alkyl}$, 34% $\beta_{asym} Ph_{alkyl}$
1435	1500	1462	99% $\delta CH3_{alkyl}$
1468	1521	1483	68% $\delta CH2_{alkoxy}$
1495	1525	1487	73% $\beta_{asym} Ph_{alkyl}$
1508	1542	1503	47% $\beta_{asym} Ph_{alkoxy}$
1600	1643	1602	71% $\beta_{sym} Ph_{alkoxy}$
1661	1742	1698	90% $\nu C=O$
2849	3012	2870	86% $\nu_{sym} CH2_{alkoxy}$
2872	3032	2889	94% $\nu_{sym} CH2_{alkoxy}$
2915	3057	2913	82% $\nu_{asym} CH2_{alkoxy}$
2956	3079	2934	84% $\nu_{asym} CH2_{alkyl}$
3041	3186	3036	96% $\nu_{asym} CH/Ph_{alkoxy}$
3049	3190	3040	94% $\nu_{asym} CH/Ph_{alkyl}$
3060	3214	3062	100% $\nu_{sym} CH/Ph_{alkoxy}$

Table S7. The band assignment of the experimental IR spectra of 11OS5 at the room temperature, based on the DFT calculations (**631+Gd** basis set, **B3LYP-D3(BJ)** functional). Notations: β – in-plane deformation, γ – out-of-plane deformation, δ – scissoring, ν – stretching, ρ – rocking, τ – twisting, ω – wagging.

experimental peak position [cm^{-1}]	unscaled calculated peak position [cm^{-1}]	scaled calculated peak position [cm^{-1}]	vibration (contribution ≥ 10%)
384	387	378	55% δCCC_{alkyl}
401	404	395	14% δCCC_{alkoxy}
416	433	423	35% νCS
445	448	438	31% δCCC_{alkoxy}
468	470	459	26% δCCC_{alkoxy}
484	492	481	27% γPh_{alkyl}
500	503	491	43% δCCC_{alkyl}
506	508	496	84% γPh_{alkoxy}
523	531	519	43% δCCO_{alkoxy}
570	577	564	21% δCCC_{alkoxy}, 13% $\beta_{asym}Ph_{alkoxy}$, 10% νCS
628	645	630	52% $\beta_{asym}Ph_{alkoxy}$, 14% δCCC_{alkoxy}
641	646	631	75% γCOS, 13% γPh_{alkoxy}
661	666	650	39% $\beta_{asym}Ph_{alkoxy}$
670	677	661	29% $\nu SC/Ph_{alkyl}$, 20% $\beta_{sym}Ph_{alkyl}$
723	736	719	73% $\rho CH_{2alkoxy}$
733	743	726	74% ρCH_{2alkyl}
750	765	747	32% ρCH_{2alkyl}, 23% γPh_{alkyl}
781	795	776	51% $\rho CH_{2alkoxy}$
803	818	799	67% γPh_{alkyl}
819	839	819	29% $\beta_{asym}Ph_{alkoxy}$, 18% νCO_{alkoxy}
839	850	830	46% γPh_{alkoxy}
851	870	850	36% γPh_{alkyl}, 21% ρCH_{2alkyl}
906	919	898	35% νCS, 26% $\beta_{asym}Ph_{alkoxy}$
934	982	959	80% γPh_{alkoxy}
946	1004	981	64% νCC_{alkoxy}
987	1029	1005	62% $\beta_{asym}Ph_{alkoxy}$, 18% νCC_{alkoxy}
1015	1051	1015	45% νCC_{alkoxy}
1023	1071	1035	68% νCC_{alkoxy}
1051	1081	1044	73% νCC_{alkoxy}
1095	1122	1084	70% $\nu SC/Ph_{alkoxy}$
1119	1154	1115	68% $\beta_{sym}Ph_{alkoxy}$
1168	1198	1157	90% $\beta_{sym}Ph_{alkoxy}$
1212	1245	1203	71% $\beta_{asym}Ph_{alkoxy}$
1231	1271	1228	53% $\omega CH_{2alkoxy}$
1269	1303	1259	52% $\nu_{asym}COC_{alkoxy}$, 12% $\beta_{asym}Ph_{alkoxy}$
1310	1363	1317	36% $\beta_{sym}Ph_{alkoxy}$, 14% ωCH_{2alkyl}
1323	1364	1318	24% ωCH_{2alkyl}, 20% $\beta_{asym}Ph_{alkyl}$
1353	1377	1330	51% $\omega CH_{2alkoxy}$
1378	1434	1385	83% ωCH_{3alkyl}
1400	1443	1394	57% $\omega CH_{2alkoxy}$
1421	1450	1401	52% $\beta_{asym}Ph_{alkyl}$, 14% ωCH_{2alkyl}
1435	1522	1470	82% δCH_{3alkyl}, 10% $\tau CH_{2alkoxy}$
1468	1539	1487	67% $\delta CH_{2alkoxy}$
1495	1541	1489	63% $\beta_{asym}Ph_{alkyl}$
1508	1557	1504	42% $\beta_{asym}Ph_{alkoxy}$
1600	1655	1599	73% $\beta_{sym}Ph_{alkoxy}$
1661	1746	1687	89% νC=O
2849	3025	2866	81% $\nu_{sym}CH_{2alkoxy}$
2872	3046	2886	95% $\nu_{sym}CH_{2alkoxy}$
2915	3071	2910	82% $\nu_{asym}CH_{2alkoxy}$
2956	3093	2930	90% $\nu_{asym}CH_{2alkoxy}$
3041	3209	3040	93% $\nu_{asym}CH/Ph_{alkyl}$
3049	3216	3047	94% $\nu_{asym}CH/Ph_{alkyl}$
3060	3233	3063	91% $\nu_{sym}CH/Ph_{alkoxy}$

Table S8. The band assignment of the experimental IR spectra of 11OS5 at the room temperature, based on the DFT calculations (**631+Gd** basis set, **BLYP-D3(BJ)** functional). Notations: β – in-plane deformation, γ – out-of-plane deformation, δ – scissoring, ν – stretching, ρ – rocking, τ – twisting, ω – wagging.

experimental peak position [cm^{-1}]	unscaled calculated peak position [cm^{-1}]	scaled calculated peak position [cm^{-1}]	vibration (contribution ≥ 10%)
384	388	393	16% δCCO_{alkoxy}, 10% νCS
401	410	416	25% $\nu_{asym}CSC/Ph_{alkyl}$, 27% δCCC_{alkyl}
416	420	426	11% δCCC_{alkyl}
445	442	448	29% δCCC_{alkoxy}
468	462	468	25% δCCC_{alkoxy}
484	489	496	80% γPh_{alkoxy}
500	497	504	50% δCCC_{alkyl}
506	507	514	40% δCCC_{alkyl}
523	527	534	35% γPh_{alkyl}, 13% δCCC_{alkoxy}
570	554	562	15% $\beta_{asym}Ph_{alkoxy}$
628	618	627	85% γPh_{alkoxy}
641	625	634	63% $\beta_{asym}Ph_{alkoxy}$
661	642	651	56% $\beta_{asym}Ph_{alkoxy}$
670	652	661	49% $\nu_{sym}CSC/Ph_{alkyl}$, 11% $\beta_{sym}Ph_{alkyl}$
723	712	722	80% γPh_{alkyl}
733	721	731	83% $\rho CH_{2alkoxy}$
750	749	759	72% ρCH_{2alkyl}
781	776	787	32% $\rho CH_{2alkoxy}$
803	796	807	55% γPh_{alkyl}
819	805	816	46% $\beta_{asym}Ph_{alkoxy}$
839	812	823	54% γPh_{alkoxy}, 12% γPh_{alkyl}
851	818	829	42% γPh_{alkyl}
906	877	889	61% $\beta_{asym}Ph_{alkoxy}$
934	931	944	44% $\tau CH_{2alkoxy}$
946	958	971	82% νCC_{alkoxy}
987	963	976	64% νCO_{alkoxy}
1015	986	990	74% νCO_{alkoxy}
1023	999	1003	64% νCC_{alkoxy}
1051	1027	1031	72% νCC_{alkoxy}
1095	1075	1079	70% $\beta_{asym}Ph_{alkyl}$
1119	1099	1103	48% νCC_{alkyl}, 21% ωCH_{2alkyl}
1168	1161	1166	76% $\beta_{sym}Ph_{alkoxy}$
1212	1203	1208	62% $\beta_{asym}Ph_{alkoxy}$
1231	1206	1211	64% $\omega CH_{2alkoxy}$
1269	1245	1250	56% $\beta_{asym}Ph_{alkoxy}$
1310	1331	1336	53% $\beta_{asym}Ph_{alkyl}$, 14% τCH_{2alkyl}
1323	1331	1336	39% $\beta_{sym}Ph_{alkoxy}$, 14% νCS, 11% $\omega CH_{2alkoxy}$
1353	1337	1342	58% $\omega CH_{2alkoxy}$
1378	1366	1372	69% $\omega CH_{2alkoxy}$
1400	1392	1398	63% $\omega CH_{2alkoxy}$
1421	1401	1407	64% $\beta_{asym}Ph_{alkyl}$
1435	1483	1489	71% $\delta CH_{2alkoxy}$
1468	1492	1498	61% $\delta CH_{2alkoxy}$
1495	1496	1502	68% $\delta CH_{2alkoxy}$
1508	1499	1505	69% $\beta_{asym}Ph_{alkoxy}$
1600	1590	1596	69% $\beta_{sym}Ph_{alkoxy}$
1661	1667	1674	87% $\nu C{=}O$
2849	2942	2864	80% $\nu_{sym}CH_{2alkoxy}$
2872	2964	2886	92% $\nu_{sym}CH_{2alkoxy}$
2915	2987	2908	81% $\nu_{asym}CH_{2alkoxy}$
2956	3009	2929	83% $\nu_{asym}CH_{2alkoxy}$
3041	3121	3038	84% $\nu_{sym}CH/Ph_{alkyl}$
3049	3135	3052	99% $\nu_{sym}CH/Ph_{alkoxy}$
3060	3148	3065	90% $\nu_{sym}CH/Ph_{alkoxy}$

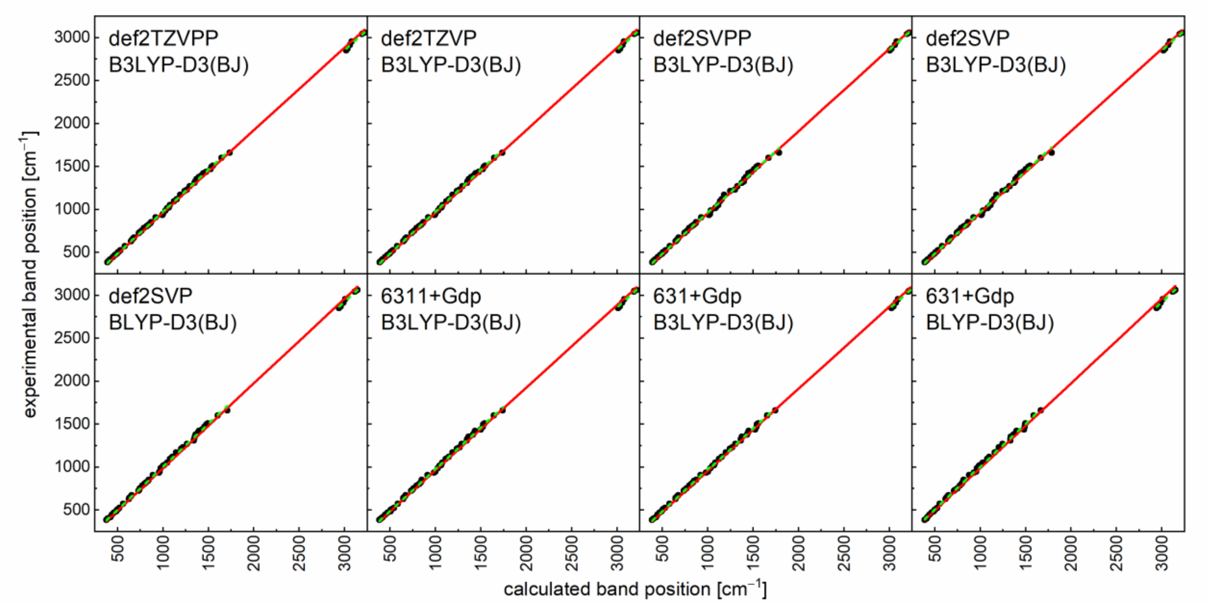

Figure S1. Experimental band positions in the IR spectrum of 11OS5 in the crystal phase in the room temperature vs. calculated band positions at different levels of theory. Solid and dashed lines indicate linear fits with intercept fixed to zero in the full spectral range and separate in the <1000 cm^{-1}, 1000-2000 cm^{-1}, >2000 cm^{-1} ranges, respectively.

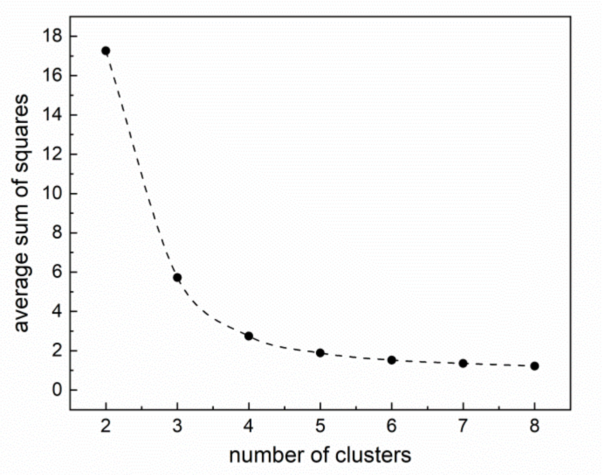

Figure S2. Determination of an optimal number of clusters in the k-means cluster analysis by the “elbow method”.
